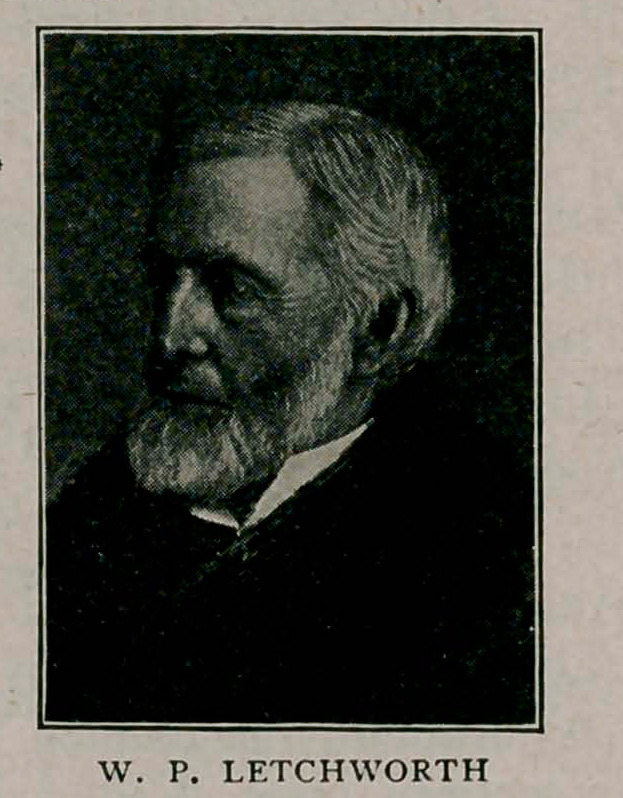# Books and Authors

**Published:** 1912-05

**Authors:** 


### BOOKS AND AUTHORS

**The Life and Work of William Pryor Letchworth,** Student and Minister of Public Benevolence, by **J. N. Larned** of Buffalo, Author of "A Study of Greatness in Men," etc. With photogravure portrait and othter illustrations. Crown 8 vo. $2.00 net. Postage 15 cents. Houghton Mifflin Company, 4 Park St., Boston, 16 E. 40th St., New York.

W. P. LETCHWORTH

Mr. Letchworth was of a type fortunately common in the old world and perhaps, fortunately rare in this country but admirable in itself—that of a gentleman of leisure, from the business standpoint, but devoting that leisure to the welfare of humanity instead of self gratification. Born in 1823, his active business life ended in 1873, when Gov. Dix of "Shoot him on the Spot" fame, appointed him to the New York State Board of Charities. On this Board, he served till 1896, being President from 1877 to 1888. He labored especially in the interests of children, to secure their material care, to bring them back from lives of vice and crime, not merely by institutionalism but by educating and appealing to the humanity of the people at large. He also interested himself in the care of the insane and the epileptic and was indirectly and ultimately, the originator of the Craig Colony for Epileptics. In the work thus taken upon himself, he built up a considerable library of his own writings. He also did much to preserve the legends, history, mementoes and part of the favorite hunting ground of the Indians and in 1906 he deeded to the State a private park of over a thousand acres, including the three falls of the Genesee near Portage.

We take pleasure in reviewing his work at some length, not merely because of local interest in the man and local appreciation of his benevolences. His sociologic accomplishments, like the more tangible gift of scenic and historic value, represented something more than the kindly inclination and financial ability to do something for his fellows and for posterity. In both regards, he exercised judgment based on long experience and careful study. As medical men, we must recognize that this layman who would have been the last person to claim expert knowledge in medical art and science, was never-the-less, in the broad problems of the care of the epileptic and the insane, himself an expert. Not merely from the accident of wealth, and official position, but because of patient study, in Europe as well as at home, because of the advocacy of well matured ideas verbally and in his writings, he has made possible strictly medical advances in the care of these unfortunates.

Mr. Larnd, from a long experience in historic study and writing, has focussed his own talents on the life story of Mr. Letchworth, so as to produce a book of interest even to the casual reader.

---

**Home Hygiene and Prevention of Disease, Norman E. Ditman,** M. D., New York. Published by Duffield & Co., 1912. 333 pages. $1.50 net.

This work is arranged alphabetically, for convenience of reference. It gives in plain language, a great deal of the symptomatology, prophylaxis and treatment, even allusions to pathology, of all of the common diseases, much sensible advice as to exercise, diet, sanitation, and even instructs somewhat in profesional ethics and ideals. The style is not noticeably condensed and it is only when one compares the scope with the size of the book, that one realizes that the author is a master of the art of brevity. The profession is somewhat prejudiced against popular medical books and especially against one that aims in any way to encourage domestic meddling with serious cases. But the physician who reads this book will find this prejudice oozing away. Not only is the author temperate in his statements and possessed of common sense, but, throughout the book he aims to train the lay reader to look at medical matters from the viewpoint of the conscientious physician and to train him—or her—not to take rash chances and to interfere, but to be intelligent and helpful.

---

**Surgical Operations,** a hand-book for Students and Practitioners, by **Prof. Pels-Leuden,** Berlin. Only authorized English translation by **Dr. Faxton E. Gardner,** of New York. Published by the Rebman Co., New York, 1912. 757 pages, 568 illustrations, cloth, $7.00.

The work, both in its execution and in the temperament of the author, is essentially practical, thus conforming rather to American than to German standards. Naturally, in many details, the reader will differ from the opinions expressed. In such cases, it is worth remembering that the author himself is abundantly justified in expressing his own opinion, and that, in various moot points, he follows—or rather agrees with—Billroth, Krause, Sauerbruch and Bauer. The Publishers are to be complimented on their part of the work. One detail we think worth mentioning . This book is about two-third the thickness and has about two-thirds of the weight of the average, glossy paper book.

---

**Operative Obstetrics,** including the **Surgery of the Newborn,** by **Edward P. Davis,** M.D., Professor of Obstetrics, Jefferson Medical College, Philadelphia. Octavo volume of 483 pages, with 264 illustrations. Philadelphia and London, W. B. Saunders Company, 1911. Cloth, $5.50 net.

This work includes operations during pregnancy, not only such as depend directly upon pregnancy, as ectopic gestation, therapeutic abortion, etc., but removal of the appendix, of diseased tubes, etc. It illustrates and treats fully, the mechanics of normal and abnormal labor, and mechanic, non-vulnerative operations such as version, application of forceps, etc. Of especial interest are the illustrations of maternal deformity leading to Caesarian section and of X-ray methods of determining or excluding foetal fractures, etc. At the end of each chapter is given a bibliography, largely from European sources. Regarding certain topics of especial rarity or interest, one would wish a more complete bibliography for historic purposes yet such compilations are probably better left for special articles than included in text books.

---

**A Manual of Pathology**, by **Guthrie McConnell**, M. D., Professor of Pathology and Bacteriology, Temple University, Medical Dept., Philadelphia. Second Revised Edition. 12mo of 531 pages, illustrated. Philadelphia and London, W. B. Saunders Company, 1911. Flexible leather, $2.50 net.

This is an excellent, condensed treatise, convenient as a pocket companion for sudents but is by no means without value for the practitioner. We would suggest to the physician of ten or more years' practice, who has become somewhat rusty on pathology, that he read through this little work, which is well systematized and does not contain a mass of detail. Not only will his recollections be refreshed and corrected, but he will be surprised to find that, every now and then, light will be thrown on a puzzling clinical case, with very practical results.

---

**Clinical Diagnosis**, a Manual of Laboratory Methods, by **James Campbell Todd**, MD., Professor of Pathology, University of Colorado. Second edition, revised and enlarged. 12mo of 469 pages with 164 text-illusutrations and 13 colored plates. Philadelphia and London, W. B. Saunders Company, 1912. Cloth $2.25 net.

The book begins with a chapter on the use of the microscope.

Sputum, urine, stomach contents, blood, faeces, are discussed systematically, with what impresses the reviewer as a fair balance of chemic and microscopic methods and due regard for the needs and limitations of the practitioner. Various miscellaneous examinations, as of pus, semen, etc., technic of investigations for animal parasites and bacteria, preparation of vaccines, etc., are added.

---

**Transaction of the 17th Annual Meeting of the American Laryngologic (al) Rhinologic (al) and Otologic (al) Society**, held at Atlantic City, June 1-3, 1911. Including meetings for the current year of the Eastern, Middle, Southern and Western sections.

We cannot attempt to review the large number of papers, covering, for the most part in symposiums, a wide range of subjects. Last month, we reviewed the Proceedings of the 33d meeting of the American Laryngologic (al) Association, held in Philadelphia in May, 1911. We know nothing of the organization of these two bodies, excepting as the work of each shows a high order of merit; we take no sides in a controversy if, indeed, there be one; but we cannot refrain from expressing the conviction that greater economy of time, money and effort and greater efficiency would result from a union of such special interests as are here represented.

**Modern Urinology.** A System of Urine Analysis and Diagnosis. Illustrated, by **Clifford Mitchell,** A. B., M.D., Professor of Chemistry, Clinical Urinology and Renal Diseases, Hahnemann Medical College, Chicago, Ill. 636 pages. Cloth, $3.00 net. Postage, 27 cents. Philadelphia, Boericke & Tafel, 1912.

This work impresses us as one of the clearest, most practical and yet most scientific that have been published. The alphabetic index of tests, following the table of contents, the list of topics at the beginning of each chapter, the assembling of information as under the color of the urine, "where the blood comes from," etc., the critical statements of advantage and disadvantages of different methods, greatly facilitate reference. Therapeutic hints, medico-legal considerations and various other practical deductions are interpolated. For instance, under the head of chlorids, we are informed that malingering in regard to the claim of starvation may be detected by the amount of chlorids, which are retained as soon as food is withdrawn.

---

**E. Merck's Annual Report of Recent Advances in Pharmaceutic (al) Chemistry and Therapeutics,** Vol. 24, **Darmstadt,** July 1911.

This work is in idiomatic English and is well worth the physician's attention. For instance, few of us realize that there are 11 available compounds of cacodylic acid and that this substance is to be seriously considered as a rival of salvarsan. The value of kaolin, internally, has been somewhat of a hobby with the editor but many will be surprised to learn that this old-fashioned remedy deserves attention. The distinction between fluorescin and fluorescein, and the preparation of the former from the latter and reconversion by oxidases, as haemoglobin, as a test, make interesting reading. There is one practical point suggested by the issue of such reports and circulars, from foreign cities to American physicians, even when the firms have American branches. Ask your postmaster why. And, when you have found out, take up the matter with your representative.

---

**Thirty-eighth Annual Report of the Secretary of the State Board of Health, Dr. Frank W. Shumway,** Lansing, Mich., 1911.

The problem of disposing of the sewage of the beet sugar plants and the statistics of small-pox, including the direction of spread of infection, are among the most interesting portions of the work. We would advise writers on the suguar industry, economics in which sugar plays an important role, sewage and vaccination, to consult this report. Health officials will, of course, receive copies.

**Money in Goats**, by **W. Sheldon Bull** of Buffalo, published by the Wakefield Press, Buffalo, 24 pages, illustrated, paper covers, 25 cents.

Physicians who have lost cases of tuberculosis when the availability of milk from an immune animal might have afforded a chance of saving life, and those who have wished in vain for the high fat content of goat's milk for certain cases of infant feeding, will be interested in the effort of Mr. Bull to popularize the goat industry. We are apt to think of the goat as an animal of minor importance. Yet this country imports thirty million dollars' worth of skins, each year, in spite of an already appreciable and constantly increasing local industry. In 1910, 115,811 goats were slaughtered in the United States for food, 226 being condemned but, of a total of over 283,000 killed in the four years, 1907-10, none were condemned for tuberculosis. Whether the natural immunity of the goat has any active value as a prophylactic or a curative agent, is subjudice. At any rate, goat's milk is a valuable commodity and the industry opens a field of employment to many persons of limited capital and strength. The mere fact that the goat is small, and cheap as compared with the cow and that the excrement is much less bulky and objectionable, renders it possible to have a supply of fresh milk in places where it would be impossible to keep a cow. And, what is important, the milk is agreeable to the palate.

---

**A Handbook of Practical Treatment,** in three volumes, by 82 eminent specialists. Edited by **John H. Musser,** M.D., Professor of Clinical Medicine, University of Pennsylvania; and **A. O. J. Kelly,** M.D., late Assistant Professor of Medicine, University of Pennsylvania. Volume III; Octavo of 1,905 pages, illustrated. Philadelphia and London, W. B. Saunders Company, 1912. Per volume: Cloth, $6.00 net; half morocco, $7.50 net.

The larger and the wider the scope, the more difficult does it become to do justice to a book. The present volume deals with constitutional, digestive, urinary, nervous, muscular and mental diseases. Like the others in the series, it enters into details, reviews long established as well as more or less novel therapeutic procedures and while necessarily reflecting the personal views of the writers, aims to do full justice to all reasonable opinions as to therapy. If we may be pardoned for a minor criticism, we don't quite like that expression "By 82 eminent specialists." It reminds us too strongly of claims of therapeutic ability that we see in the daily press. A considerable number of the writers are not specialists at all in the accepted professional sense and they are all too eminent to wish to have the term applied.

**Annual Report of the Surgeon-General of the Public Health and Marine Hospital Service of the U. S.,** for the year 1911.

While this is largely a business report, scientific work being alluded to only statistically, there are many points of extreme interest. A beginning has been made on the investigation of the water of the Great Lakes which, it is estimated, will be depended on by a quarter of the population of the country, within a few years. Small-pox, anti-typhoid vaccination, pellagra, cholera, epidemic meningitis, are among the big problems of this service, in addition to the routine care of revenue and merchant marine sailors. Curiously enough this service has only 135 commissioned officers and employs 283 acting assistant surgeons. It would seem—but no, let us attend to our own business and not try to offer advice without full undertanding of the conditions.

---

Battle & Co. have just issued No. 8 of the Dislocation Charts which completes the set. They will be sent free to Physicians on request. Also Fracture and Tumor charts if desired.

## Figures and Tables

**Figure f1:**